# Editorial: Women offenders: the challenge of evidence-based practice in correctional and forensic mental health services

**DOI:** 10.3389/fpsyt.2026.1938724

**Published:** 2026-09-08

**Authors:** Juliane Gerth, Caroline Logan, Irina Franke, Tammi Walker

**Affiliations:** 1 Department of Psychology, Universitat Konstanz, Konstanz, Germany; 2 Department of Research and Development, Office of Corrections and Rehabilitation, Canton of Zurich, Switzerland; 3 Department of Security and Crime Science, University College London, London, United Kingdom; 4 Department of Forensic Psychiatry, Psychiatric Services of Grisons, Cazis/Chur, Switzerland; 5 Department of Forensic Psychiatry and Psychotherapy, Ulm University, Ulm, Germany; 6 Department of Psychology, Durham University, Durham, United Kingdom

**Keywords:** female offenders, forensic mental health, gender-sensitive practice, justice-involved women, risk assessment and treatment, trauma-informed care

Women are a minority of those held in prisons and forensic mental health services, and this numerical imbalance has constrained the evidence base available to those who care for them. Because populations are small, research remains limited; as a result, practice has often been borrowed wholesale and unadapted from studies conducted with boys and men. As D’Orta et al. note, forensic research on women was for decades built on the assumption that findings from research into men and boys could be applied to women and girls without change. Yet, the contributions to this Research Topic, drawn from jurisdictions across three continents, advance a shared argument consistent with a growing body of research: women and girls are not simply a smaller version of the male offender, but a group whose needs, pathways and risks require evidence of their own. Building that evidence is both necessary and possible, and this Research Topic contributes to that effort.

The reliance on male-derived evidence is apparent at the earliest stages of practice, including how women are selected for treatment. O’Meara et al. examine a specialist program in England and Wales for offenders with complex personality-related needs. The criteria for admitting women relied on practitioners’ judgement and a range of screening items developed for men. Using women’s clinical and case records, the authors derive a sixteen-item, gender-sensitive screening tool that better reflects women’s psychological needs while maintaining stable admission rates. The same pattern appears in prescribing. In a Geneva forensic unit, D’Orta et al. found that second-generation antipsychotics were prescribed to 77% of admitted women, while fewer than a third had a diagnosed psychotic disorder, highlighting a mismatch between diagnosis and prescribing. Dosage appeared to reflect psychiatric history more than current diagnosis. While prescribing to manage acute crises and complex symptom presentations has a clear rationale, off-label prescribing on this scale warrants closer scrutiny.

Risk assessment, too, has lacked a robust foundation, as appropriate reference data for women has been scarce. Mayer et al. address this gap by providing rare offence- and disorder-related reconviction rates for women discharged from German forensic care. Treatment, they find, lowers both the rate and the severity of reoffending; comorbid disorders raise the risk; and forensic aftercare is central to preventing it — a foundation for the development of gender-responsive risk assessment, which remains underdeveloped for women. At each step — screening, prescribing and risk prediction — practice for women rests on foundations largely built for men, or only partially developed.

If the male template is limited, what should evidence-based practice for women take into account? Several contributions address this question. Comparing thirty years of admissions to one English service, McCarthy et al. find that women entering specialized single-sex care present with more complex criminological and trauma histories — more self-harm, alcohol use and childhood adversity — than the earlier mixed-sex cohort. Self-harm, which is substantially more prevalent in women’s custody than in men’s, is the focus of the contribution by Robinson et al., who bring an economic perspective to a problem usually framed only clinically: their collaboratively developed Prison Data Inventory captures the costs of self-harm, providing a transparent, practice-oriented basis for developing and evaluating interventions. Motherhood, which affects women in custody in ways it does not affect men, is examined by Clark et al., whose systematic review concludes that justice-involved mothers are best supported through trauma-informed, family-oriented programming rather than parenting skills taught in isolation — approaches that help them rebuild their maternal identity and foster secure attachments with their children.

Such needs are relational and situated in lived experience. Therefore, from Sweden, Revelj et al. give voice to caregivers, who describe care for women as relationship-based and grounded in sustained interpersonal engagement, yet shadowed by self-directed violence and the discomfort of coercion — restraint that staff themselves experienced as a form of assault. Their account points to a training need: helping staff strike a balance between relational caregiving and the coercive measures that managing risk may require. And from Kobo Prison in Ethiopia, Asmare et al. examine women’s own perspectives on incarceration. The women in their study describe missing mental-health support, childcare and protection from gender-based violence; yet even the limited, informal counselling and skill-building available to them emerges as a source of resilience, underscoring both unmet need and existing strengths.

Distinct needs call for distinct services, yet provision remains uneven. Páv et al., comparing 85 women with 753 men across Czech forensic facilities, find women were admitted with greater acuity — more psychotic and substance-use disorders, more violent index offences — yet were discharged sooner and placed in lower security. Páv et al. interpret this as a structural problem: with no dedicated units for women, they are placed on general wards rather than in services designed for them. McCarthy et al., by contrast, illustrate what specialized provision can achieve, describing longer stays in care built around women’s needs. Together, the two point in the same direction: where services are shaped around women, outcomes improve; where they are not, substantial need goes unmet.

That this evidence can be built at all, for a group too small for conventional trial designs, is itself an important contribution to the field. The studies gathered in this Research Topic on women offenders demonstrate a range of methodological approaches suited to this challenge, including systematic review, qualitative inquiry, secondary-data modelling, single-site cohorts, and the collaborative design of new measurement tools. The proposal that launched this Research Topic also named non-binary and transgender people, but none of the contributions addresses this cohort — a reminder that, for these groups, the evidence base remains limited and often relies on clinical consensus rather than empirical research, making this a clear priority for future work. The research collected here does not finish the task of making practice with women evidence-based. But it contributes to a growing body of research that is steadily shifting the field — away from practice borrowed from men and toward the development of genuinely gender-responsive, evidence-based approaches tailored to women’s needs.

